# Size‐Dependent Efficacy of Lipid Nanoparticles in Improving Glucose Utilization of Largemouth Bass (*Micropterus salmoides*) Under High‐Glucose Conditions

**DOI:** 10.1155/anu/7025461

**Published:** 2026-05-27

**Authors:** Kaipeng Zhang, Tengfei Zhu, Yamin Wang, Jing Chen, Shan Xie, Zhenye Lin, Xiaotong Chen, Yingying Yu, Yining Xu

**Affiliations:** ^1^ School of Animal Science and Technology, Guangdong Provincial Key Laboratory of Animal Molecular Design and Precise Breeding, Foshan University, Foshan, 528225, Guangdong, China, fosu.edu.cn; ^2^ South China Sea Fisheries Research Institute, Key Laboratory of South China Sea Fishery Resources Exploitation and Utilization, Ministry of Agriculture and Rural Affairs, Chinese Academy of Fishery Sciences, Guangzhou, 510300, Guangdong, China, cafs.ac.cn; ^3^ Sanya Tropical Fisheries Research Institute, Sanya, 572018, Hainan, China; ^4^ Department of Pharmacy, Institute of Metabolic Diseases and Pharmacotherapy, West China Hospital, Sichuan University, Chengdu, 610041, Sichuan, China, scu.edu.cn; ^5^ Department of Clinical Pharmacy and Pharmacy Administration, Laboratory of Drug-Targeting and Drug Delivery System of the Ministry of Education, West China School of Pharmacy, Sichuan University, Chengdu, 610041, Sichuan, China, scu.edu.cn

**Keywords:** aquaculture production, feed additives, gene expression, glucose metabolism, high carbohydrate feed, largemouth bass, nanotechnology

## Abstract

**Backgrounds:**

Largemouth bass (*Micropterus salmoides*) is an economically important aquaculture species, but its pronounced intolerance to high‐carbohydrate diets often leads to metabolic liver disorders and compromised growth, limiting the practical application of cost‐effective high‐carbohydrate feeds in aquaculture. Lipid nanoparticles (LNPs) have shown promise in regulating glucose metabolism in mammals, but their efficacy and size‐dependent effects in fish remain unelucidated.

**Objective:**

LNPs were evaluated as novel potential additives to enhance the glucose utilization in *M. salmoides* under high‐glucose (HG) feeding conditions, addressing the issues of glucose intolerance and thereby reducing breeding costs.

**Methods:**

We constructed LNPs to serve as a feed additive for modulating the glucose metabolism in *M. salmoides*. We prepared LNPs with five different particle sizes (60, 125, 150, 175, and 200 nm) by varying the formulation ratio of the preparation. To obtain further results, the regulatory effect of LNPs on glucose metabolism in *M. salmoides* was verified through gavage administration. The expression levels of genes associated with glucose metabolism in *M. salmoides* were observed following acute (one administration) treatment with HG feeding to investigate the relevance between LNPs and glucose metabolism induced by HG in *M. salmoides*.

**Results:**

HG exposure activated the hepatic PI3K/AKT pathway, upregulated the expression of gluconeogenesis‐related genes (*foxo1* and *g6pase*), and disrupted the expression of glycogenesis‐related genes (*gsk3* and *gys1*), leading to impaired glucose metabolism in *M. salmoides*—a key pathogenesis of high‐carbohydrate‐induced metabolic disorders. Notably, LNPs with 150–200 nm sizes specifically upregulated intestinal *glut5* mRNA expression (but not *glut2*), intestinal GLUT5 is a critical hexose transporter: traditionally recognized for mediating fructose transport, it also facilitates glucose crossing the intestinal epithelial barrier to enter the bloodstream, providing the foundation for subsequent systemic glucose metabolism, and 150 nm LNPs exhibited the most rapid efficacy: they modulated the hyperactivated PI3K/AKT pathway, reversed the abnormal expression of *foxo1*, *g6pase*, *gsk3*, and *gys1* induced by HG, and improved insulin sensitivity by upregulating *irs1* and *insr* mRNA levels.

**Conclusion:**

150 nm LNPs effectively ameliorate high‐carbohydrate‐induced metabolic damage by targeting GLUT5 to enhance intestinal glucose absorption and regulating the PI3K/AKT‐gluconeogenesis/glycogenesis cascade, providing a targeted solution for improving HG feed utilization in carnivorous fish aquaculture.

**Summary**

• Lipid nanoparticles (LNPs) affect the intestinal transit process in largemouth bass.
• LNPs can improve the glucose utilization ability of largemouth bass.
• The impact of LNPs on glucose utilization correlates with their particle size.

## 1. Introduction

Largemouth bass (*Micropterus salmoides*) is an economically important carnivorous fish, prized for its fast growth and desirable flesh quality. In modern aquaculture, there is a growing trend to incorporate higher levels of dietary carbohydrates as a cost‐effective strategy to reduce dependence on expensive fish meal [1, 2]. While such high‐carbohydrate diets can spare protein, carnivorous fish like *M. salmoides* exhibit a well‐documented intolerance to excessive carbohydrates, often resulting in persistent hyperglycemia, hepatic glycogen and lipid accumulation, and metabolic liver disease. [3–5]. These metabolic disturbances are linked to dysregulation of key pathways such as hepatic PI3K/AKT, a central regulator of insulin‐mediated glucose homeostasis, signaling, and elevated gluconeogenic gene expression (*foxo1*, *pepck*, and *g6pase*), ultimately impairing glucose homeostasis [6]. Therefore, developing effective interventions to enhance carbohydrate utilization in *M. salmoides* remains a critical need for sustainable aquaculture.

Recent advances in nanonutrition suggest that lipid nanoparticles (LNPs) could offer a novel approach to modulate glucose metabolism. In murine models, certain LNPs (50–200 nm) can mimic endogenous lipid ligands and stimulate the secretion of glucagon‐like peptide‐1 (GLP‐1), a hormone that improves glucose tolerance and insulin sensitivity [7–9]. GLP‐1 or its analogs are known to enhance insulin sensitivity by activating the hepatic PI3K/AKT signaling pathway, which in turn leads to the phosphorylation and inactivation of the forkhead transcription factor FoxO1 [10]. The inhibition of FoxO1 is a key event that mediates the downstream transcriptional downregulation of critical gluconeogenic genes, such as *pepck* and *g6pase*, thereby suppressing excessive glucose production and contributing to improved glucose homeostasis [11, 12]. However, whether LNPs can exert similar regulatory effects in fish—especially under high‐glucose (HG) conditions—has not been explored. Moreover, the functionality of nanoparticles depends critically on their cellular uptake and transport, which are strongly influenced by particle size [13, 14].

Based on this background, we hypothesize that LNPs of specific sizes may enhance intestinal transport and hepatic glucose metabolism in *M. salmoides* under high dietary carbohydrate stress. To test this, the present study was designed to: (1) evaluate the effects of LNPs with different particle sizes on intestinal uptake and transport capacity; (2) assess their ability to ameliorate HG‐induced metabolic dysregulation in the liver; and (3) elucidate the underlying molecular mechanisms. Our work aims to provide the first evidence for the potential of LNPs as a novel nano‐additive to improve glucose utilization in largemouth bass, offering a new strategy to mitigate carbohydrate‐induced metabolic disorders in carnivorous fish aquaculture.

## 2. Materials and Methods

### 2.1. Formulation and Characterization of the LNPs

#### 2.1.1. Preparation of LNPs With Different Particle Sizes

Lipoid S100 (soya phosphatidylcholine), a light‐yellow powder with high purity (≥94% phosphatidylcholine), was purchased from Lipoid GmbH (Ludwigshafen, Germany). Labrafac WL 1349 (medium‐chain triglycerides, mainly from coconut oil), a colorless to pale yellow liquid, was obtained from TianRun Pharmaceutical Co., Ltd. (Guangzhou, China). Solutol HS15 (polyethylene glycol 660 hydroxystearate), a white viscous liquid (melting point~30°C), was sourced from Sigma–Aldrich (St. Louis, MO, USA). Sodium chloride (NaCl), a white crystalline powder of analytical grade, was supplied by Sinopharm Chemical Reagent Co., Ltd. (Ningbo, China).

A reversed‐phase method was used to prepare LNPs with sizes closely related to different excipient ratios. In brief, a mixture was prepared by combining NaCl, Labrafac WL 1349, Lipoid S100, lipophilic Solutol HS15, and ultrapure water as the constituents at 40°C. Three alternating thermal processes of gradual heating and cooling (from 60 to 90°C) were performed. In the last temperature cycle, 1.2 mL/g cold water (4°C) was added in the cooling step with high‐speed stirring at 74°C (Table 1). The resulting LNPs were filtered through a 0.45 μm filter and subsequently stored at 4°C. Different proportions of water, surfactant, and oil were adjusted in the formulation to prepare LNPs with different particle sizes. Increasing the oil phase in the formulation was able to increase the particle size, conversely, increasing the surfactant reduced the particle size (Table 2) [7, 15]. The concentration of LNPs (g/L): (W_Labrafac WL 1349_ + W_HS15_ +W_Lipoid S100_) /V_LNPs_.

**Table 1:** Preparation process of lipid nanoparticles.

Procedure	Temperature (°C)	Speed (rpm)	Time (min)	Notic
a	40	350	5	/
b	90	350	5	/
c	60	350	5	Slowly cooling
d	90	350	5	/
e	60	350	5	Slowly cooling
f	90	350	5	/
g	77–79	350	3	Add water (4°C)

**Table 2:** Composition of LNPs with different particle sizes.

Size (nm)	60	125	150	175	200
Solutol HS15 (g)	0.846	0.580	0.532	0.290	0.250
Lipid S100 (g)	0.075	0.075	0.075	0.075	0.075
Labrafac WL1349 (g)	1.028	1.112	1.257	1.789	2.000
NaCl (g)	0.089	0.089	0.089	0.089	0.089
Water (25°C) (mL)	2.962	3.143	3.047	2.756	2.586
Water (0°C) (mL)	6.000	6.000	6.000	6.000	6.000
Concentration (g/mL)	0.177	0.161	0.169	0.196	0.211

#### 2.1.2. Characterization and Stability of LNPs

The LNPs’ particle size were measured using dynamic light scattering (DLS) with a Zetasizer Nano ZS (Malvern Instruments Ltd., Worcestershire, UK), according to ISO 22412:2017. The same device was used to measure the zeta potential of the lipid nanoemulsion, equipped with a 4 mW He/Ne laser generator emitting at a wavelength of 633 nm. The zeta potential of the lipid nanoemulsion was determined by applying an electric field to both ends, as per the experimental protocol [7].

The resulting LNPs were stored at 4 and 25°C for 30 days to observe their stability.

### 2.2. Ethical Statement

All experiments conducted in this study received approval from the Foshan University Animal Ethics Committee (Approval Number: 2020056) and executed guidelines for the National Institutes of Health Guide for the Care and Use of Laboratory Animals of China.

### 2.3. Animals, Acclimation, and Husbandry

A total of 415 juvenile largemouth bass (*M. salmoides*) were obtained from Sanshui Baijin Aquatic Seedling Co., Ltd. (Foshan, China). Fish were acclimatized for 2 weeks in a recirculating aquaculture system while fed a commercial diet (Fenghua Perch Compound Feed) twice daily. From this stock, 378 individuals (12.14 ± 0.57 g) were selected for the experiment. Water quality was maintained within optimal ranges: dissolved oxygen >7.0 mg/L, pH 7.5–8.5, temperature 24 ± 1°C, and ammonium nitrogen (NH_4_‐N) <0.3 mg/L, with continuous aeration.

### 2.4. Experimental Design and Grouping

The fish were randomly allotted into seven treatment groups (*n* = 54 per group), each with three tank replicates (18 fish per 70 L tank): Control: administered 200 µL of sterile saline; HG: administered 100 µL of glucose solution; HG60, HG125, HG150, HG175, and HG200: administered 100 µL of glucose solution followed by 100 µL of LNPs with mean diameters of 60, 125, 150, 175, and 200 nm, respectively.

### 2.5. Gavage Procedure

After 24 h of fasting, fish were anesthetized via ice–water immersion until opercular movement slowed significantly. Oral gavage was performed using a 200 µL Hamilton syringe fitted with a sterile, flexible polyethylene catheter (outer diameter: 0.9 mm). The catheter was gently inserted into the mouth and advanced along the upper palate, through the esophagus, and into the stomach. For fish of this size (mean weight 12.14 ± 0.57 g), the catheter was advanced to a standardized depth of 2.0–2.5 cm from the mouth opening to ensure consistent intragastric delivery. The solutions were administered by depressing the plunger slowly and steadily over a period of 2–3 s. After gavage, fish were held in water for a brief observation period to confirm no immediate regurgitation before being returned to their tanks. Fish in the HG and all LNP groups received a glucose solution at 1.67 g/kg body weight [16]. Immediately thereafter, fish in the LNP groups received a suspension of LNPs at 500 µg/kg body weight [9]. Control fish received an equivalent volume of saline.

### 2.6. Sample Collection

At 0, 30, 60, 120, 180, and 240 min postgavage, three fish per replicate tank (*n* = 9 per treatment per time point) were euthanized. Liver tissues were excised, immediately snap‐frozen in liquid nitrogen, and stored at –80°C for subsequent RNA extraction and quantitative real‐time polymerase reaction (qPCR) analysis.

### 2.7. qPCR

Relevant liver samples (three liver samples per replicate) were systematically collected and promptly cryopreserved in liquid nitrogen for subsequent RNA extraction. The isolation of total RNA was performed using the RNA preparation kit (TransGen Biotech Co., Ltd., Beijin, China) and reverse‐transcribed using a cDNA synthesis kit (TransGen Biotech Co., Ltd., Beijin, China). The test was performed three times by SYBP Green Master Mix (Yeasen Biotechnology Co., Ltd., Shanghai, China) to perform the expression of selected mRNAs in an Applied Biosystems QuantStudioTM Real‐Time PCR system (QuantStudio5, Thermo Fisher Scientific, Massachusetts, USA). Reaction procedure: predenaturation was performed at 95°C for 30 s, followed by 40 cycles of denaturation at 95°C for 3 s and annealing at 60°C for 20 s. The *β-actin* (forward sequence (5^′^–3^′^): AAAGGGAAATCGTGCGTGAC; reverse sequence (5^′^–3^′^): AAGGAAGGCTGGAAGAGGG) was employed as the reference gene. The primers for qPCR were designed according to the nucleotide sequences of *M. salmoides* target genes (Table 3). The expression of genes was counted and standardized used the 2^−ΔΔCT^ methods [17].

**Table 3:** Primers used in the present study.

Gene	Forward sequence (5^′^–3^′^)	Reverse sequence (5^′^–3^′^)	Produce length (bp)
*β-actin*	AAAGGGAAATCGTGCGTGAC	AAGGAAGGCTGGAAGAGGG	184
*akt*	GCCGTTCTACAACCAGG	TGAGGCTTGAAGGGAGG	243
*fbp1*	GCGATTGGCGAATTTATC	ACTCTGTGACGGCGGGTT	112
*foxo1*	AGGAGACCGAGGACTTTACG	TGATGATGCGGGAGTTGC	166
*g6pase*	ACTATGGAAGAGAACCGGCC	TACCATCACGTACCAGACCG	116
*gcgr*	TGTGCCATGGGTTGTAGCTA	ATTTGGTGCGCTCGAAGTTT	177
*gck*	GGGTTTTACCTTCTCCTTTC	GGTGGCTACTGTGTCATTCA	190
*glut2*	GTGTTTGCTGTGCTGCTCCT	GCTCCGTATCGTCTTTGGG	145
*gsk3*	CATCGGTAATGGCTCGTTCG	TCTGCAGCTCACGGTTCTTA	116
*gys1*	TTATTCTCTCTCGCTCCCGC	ATCTGCCTATCACCTGCCTC	151
*insr*	ACCCCAAGAGAGATGTCGAC	GCCTCGCTTCACCATCATTT	134
*irs1*	AGGTGGATGACTCTGTGGTG	CTCGGTGGCAGATTTGGATG	184
*pfk*	TACTGAACTGAAGGCTGCGA	GAGCGTCGAGTCACATGTTC	153
*pi3k*	AAGACCTTCCTCATCACGAC	CCTTCCACTACAACACTGCA	154

*Note: akt*, serine/threonine kinase 1; *foxo1*, forkhead box o1; *g6pase*, glucose‐6‐phosphatase catalytic subunit 1; *pfk*, 6‐phosphofructo‐2‐kinase/fructose‐2,6‐biphosphatase 1; *pi3k*, phosphatidylinositol‐3‐kinase p85.

Abbreviations: *fbp1*, fructose‐1,6‐bisphosphatase 1; *gcgr*, glucagon receptor; *gck*, glucokinase; *glut2*, glucose transporter type 2; *gsk3*, glycogen synthase kinase 3; *gys1*, glycogen synthase 1; *insr*, insulin receptor; *irs1*, insulin receptor substrate 1.

### 2.8. Statistical Analysis

All experimental data were analyzed using SPSS 26.0 software (IBM, Armonk, NY, USA). Continuous data were checked for normality and homogeneity of variance prior to analysis. For comparisons involving only two experimental groups, an independent two‐sample Student’s *t*‐test was applied, with statistical significance set at *p*  < 0.05 [18]. When more than two groups were compared, one‐way analysis of variance (ANOVA) was conducted first. If a significant overall effect was detected (*p* < 0.05), Duncan’s new multiple range test was used for post hoc pairwise comparisons to control the Type I error rate [19]. Results from multiple group comparisons are graphically represented, with bars sharing no common lowercase letter (a, b, c, etc.) being significantly different (*p* < 0.05). All measurements were performed with three independent biological replicates per group, and each sample was analyzed in triplicate (*n* = 9). Data in figures and tables are presented as mean ± standard deviation (SD).

## 3. Results

### 3.1. Characterization of LNPs

LNPs with five target sizes (60, 125, 150, 175, and 200 nm) were successfully prepared by adjusting formulation ratios (Tables 2 and 4). All formulations were homogeneous, milky‐white, and physically stable over 30 days at 4 and 25°C (Figure S1).

**Table 4:** Size, PDI and zeta potential of the different LNPs (mean ± SEM, *n* = 3).

Parameter	60	125	150	175	200
Size (nm)	58.87 ± 0.40	124.2 ± 0.83	149.5 ± 1.338	175.4 ± 1.222	202.1 ± 0.932
PDI	0.0709 ± 0.01	0.2388 ± 0.02	0.0547 ± 0.02	0.1919 ± 0.01	0.0847 ± 0.01
Zeta (mV)	−5.00 ± 1.52	−5.423 ± 0.47	−4.886 ± 0.57	−3.403 ± 0.22	−5.162 ± 0.73

### 3.2. Expression of Intestinal Transporters‐Related Genes

Following glucose gavage, intestinal *glut2* mRNA was upregulated in all treatment groups (Figure 1D). In contrast, the expression of *glut5*, *pi3k*, and *akt* was significantly increased only in the groups receiving larger LNPs (150, 175, and 200 nm), but not in the Con, HG, HG60, or HG125 groups (Figure 1A‐C).

Figure 1The intestinal relative expression levels of *glut5* (A), *pi3k* (B), *akt* (C), and glut2 (D) mRNA in *M. salmoides*. The distinct symbols (a, b, and c) on the lines indicate statistically significant differences between the groups (Duncan’s test, *n* = 9; *p* < 0.05).

**Figure:**
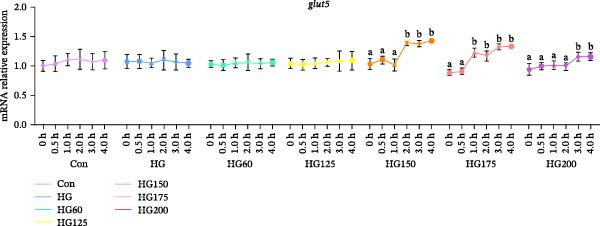
(A)

**Figure:**
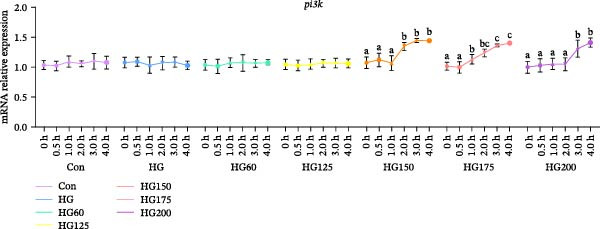
(B)

**Figure:**
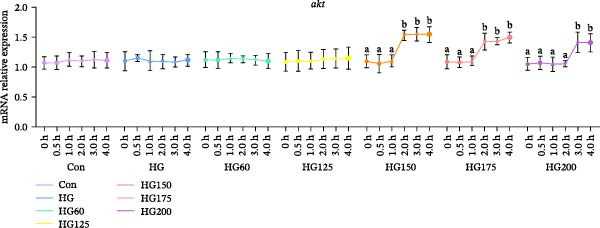
(C)

**Figure:**
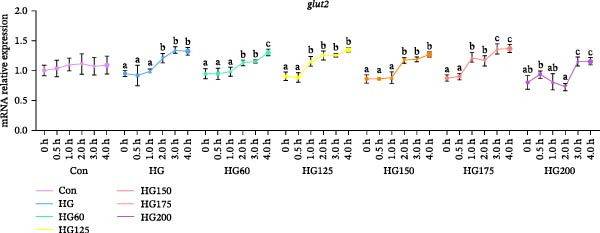
(D)

### 3.3. Expression of Hepatic Insulin Pathway and Glucose Transport‐Related Genes

The expression of hepatic *irs1* mRNA was significantly downregulated in the HG, HG60, HG125, HG175, and HG200 groups at 2 h postgavage and thereafter. Notably, this downregulation was reversed in the HG150 group, where *irs1* expression increased (Figure 2A). The expression of *insr* mRNA was significantly upregulated at 4 h in the HG150, HG175, and HG200 groups, but remained unchanged in the HG, HG60, and HG125 groups (Figure 2B).

Figure 2The relative expression levels of insulin pathway‐related genes, *irs1* (A) and *insr* (B), in the liver. The distinct symbols (a and b) on the lines indicate statistically significant differences between the groups (Duncan’s test, *n* = 9; *p* < 0.05).

**Figure:**
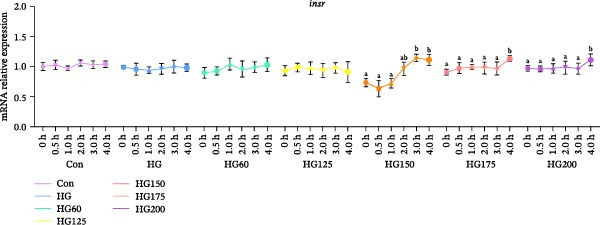
(A)

**Figure:**
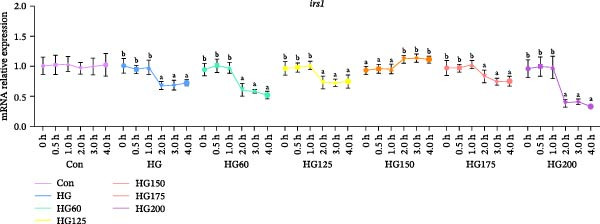
(B)

### 3.4. Expression of Hepatic PI3K/AKT Signaling Pathway‐Related Genes

HG intake activated the hepatic PI3K/AKT pathway, as evidenced by significantly increased *pi3k* and *akt* mRNA levels in the HG, HG60, HG125, and HG200 groups at 120 min (Figure 3A,B). This activation pattern was modulated by specific LNPs: the HG150 and HG175 groups showed distinct temporal expression dynamics for *pi3k* and *akt*, with the HG150 group exhibiting no significant fluctuation in *akt* expression (Figure 3A,B).

Figure 3The liver relative expression levels of *pi3k* (A) and *akt* (B) mRNA in *M. salmoides*. The distinct symbols (a, b, and c) on the lines indicate statistically significant differences between the groups (Duncan’s test, *n* = 9; *p* < 0.05).

**Figure:**
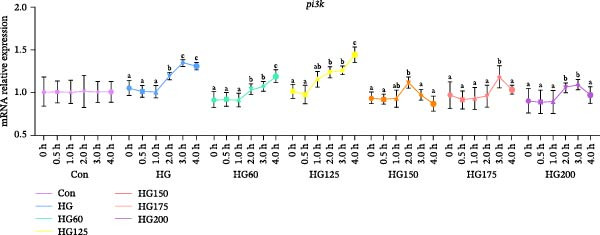
(A)

**Figure:**
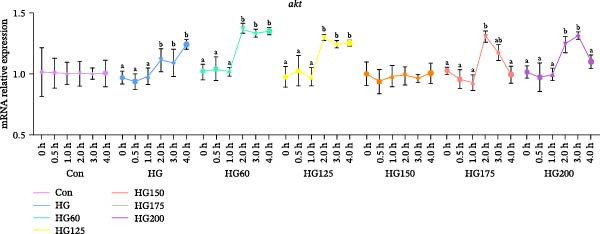
(B)

### 3.5. Expression of Hepatic Gluconeogenesis‐Related Genes

HG promoted hepatic gluconeogenesis, marked by upregulated *foxo1* and *g6pase* mRNA in the HG group and most LNP‐treated groups (Figure 4A,B). Administration of 150 nm LNPs (HG150 group) effectively suppressed this glucose‐induced upregulation, maintaining *foxo1* and *g6pase* expression levels comparable to the control (Figure 4A,B).

Figure 4The relative expression levels of gluconeogenesis‐related genes, *foxo1* (A) and *g6pase* (B), in the liver. The distinct symbols (a, b, and c) on the lines indicate statistically significant differences between the groups (Duncan’s test, *n* = 9; *p* < 0.05).

**Figure:**
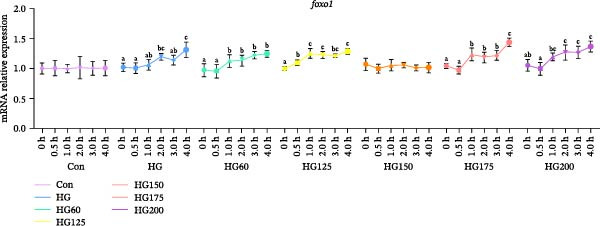
(A)

**Figure:**
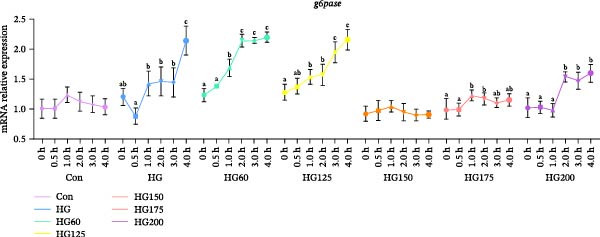
(B)

### 3.6. Expression of Hepatic Glycogen Synthesis‐Related Genes

Compared to the control, the expression of *gsk3* and *gys1* mRNA was significantly inhibited in all groups except HG150, suggesting a promotion of glycogen synthesis (Figure 5A,B). In the HG150 group, the expression of these genes remained stable, indicating that 150 nm LNPs counteracted the HG effect on glycogen synthesis (Figure 5A,B).

Figure 5The relative expression levels of glycogen synthesis‐related genes, *gsk3* (A) and *gys1* (B), in the liver. The distinct symbols (a, b, and c) on the lines indicate statistically significant differences between the groups (Duncan’s test, *n* = 9; *p* < 0.05).

**Figure:**
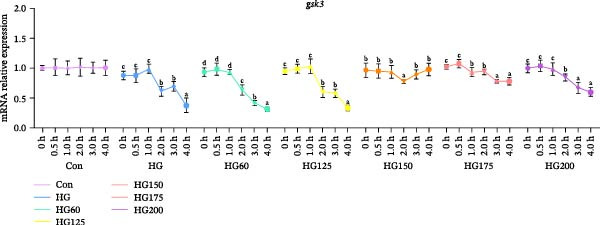
(A)

**Figure:**
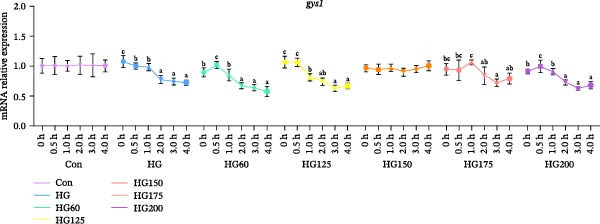
(B)

### 3.7. Expression of Hepatic Glycolytic Pathway‐Related Genes

Hepatic *glut2* mRNA expression was upregulated in all glucose‐challenged groups (Figure 6A). The expression of two key glycolytic enzymes, *gck* and *pfk*, was significantly increased within 4 h postgavage in all treatment groups, including HG (Figure 6B,C).

Figure 6The relative expression levels of glycolytic pathway‐related genes *glut2* (A), *pfk* (B), and *gck* (C) in the liver. The distinct symbols (a, b, and c) on the lines indicate statistically significant differences between the groups (Duncan’s test, *n* = 9; *p* < 0.05).

**Figure:**
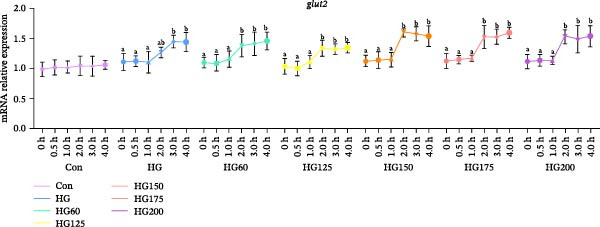
(A)

**Figure:**
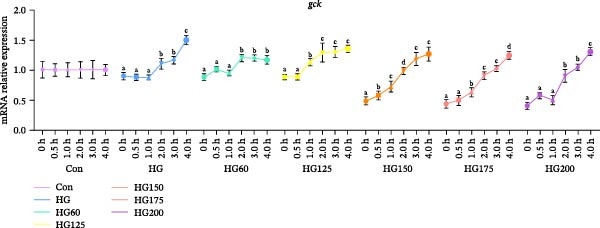
(B)

**Figure:**
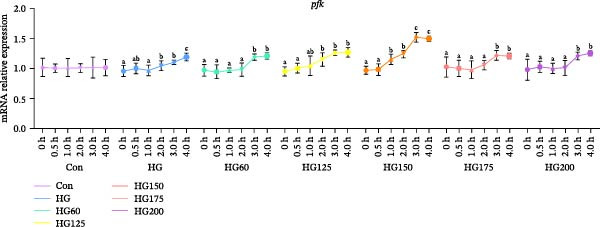
(C)

## 4. Discussion

### 4.1. Effects of LNPs of Different Sizes on Intestinal Transport in *M. salmoides*

GLUT2 and GLUT5 are crucial transporters for intestinal hexose absorption [20]. As anticipated, HG significantly upregulated intestinal *glut2* expression across all treatment groups, consistent with its role in mediating dietary glucose uptake [21]. Interestingly, while glucose alone did not alter *glut5* expression—a transporter more sensitive to fructose [22]—its mRNA levels were markedly increased specifically by LNPs in the 150–200 nm range. This suggests that these larger LNPs may influence intestinal glucose sensing or signaling through a GLUT5‐associated pathway, rather than directly competing for GLUT2‐mediated transport [23].

GLUT5 is not merely a fructose transporter; its expression can be modulated by broader nutritional and cellular states [24, 25]. Therefore, we propose that the upregulation of intestinal *glut5* observed here is unlikely to reflect a direct involvement in glucose translocation. This implies that the larger LNPs might influence intestinal glucose sensing or postabsorptive metabolic routing through a GLUT5‐associated signaling axis, rather than directly at the transport level. The precise molecular link between LNP uptake, *glut5* transcription, and downstream metabolic outcomes warrants further investigation [26].

### 4.2. Effects of LNPs of Different Sizes on Glucose Metabolism in *M. salmoides*

**Figure 7:**
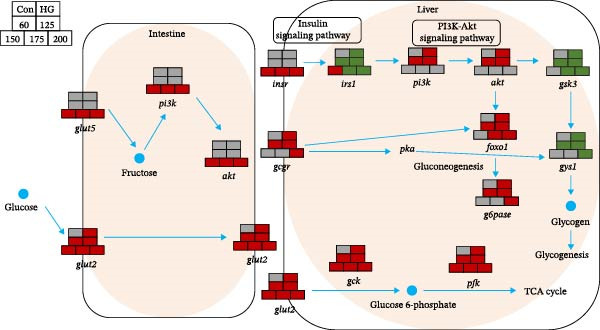
Gene interaction diagram. Each module in the figure represents a gene, and each module is composed of seven blocks, namely the Con group, the HG group, the HG60 group, the HG125 group, the HG150 group, the HG175 group, and the HG200 group (from left to right and top to bottom). Red indicates a significant upward trend of the gene within 4 h, green indicates that the gene is significantly downregulated within 4 h, and gray indicates that there is no significant change trend of the gene within 4 h. (Duncan’s test, *n* = 9; *p* < 0.05).

In mammals, the in vivo efficacy of LNPs is known to be size‐dependent, with 200 nm LNPs shown to promote GLP‐1 secretion and improve glucose metabolism [9]. This size dependency is largely governed by cellular uptake mechanisms; for instance, particles below 200 nm are primarily internalized via clathrin‐coated pits, whereas very small particles (<100 nm) can disrupt the plasma membrane due to enhanced lipid interactions [27, 28]. To determine if a similar size‐dependent effect influences glucose metabolism in *M. salmoides*, we administered LNPs of various sizes via oral gavage following a glucose challenge and assessed key metabolic genes. Our results revealed that 150 and 175 nm LNPs significantly inhibited the glucose‐induced upregulation of *GCGR* mRNA. Among all formulations, 150 nm LNPs most effectively improved overall glucose metabolism, while 175 nm LNPs specifically modulated hepatic glycogen turnover. Notably, the effects of 175 nm LNPs on glycogen metabolism differed from those observed in rats [9], highlighting potential interspecies variations in LNP activity Figure 7.

### 4.3. LNPs Improved Insulin Sensitivity in *M. salmoides*

Given that glucose peaks between 1–4 h in carnivorous fish [29], the present study examined changes in genes associated with glucose metabolism in *M. salmoides* within 4 h after glucose gavage. *M. salmoides* exhibits a limited capacity for high carbohydrate utilization [30], potentially leading to adverse effects when subjected to a diet rich in carbohydrates [31]. As this limitation may arise from inadequate insulin secretion [32], enhancing insulin sensitivity could enhance the utilization of carbohydrates [33]. As a new drug delivery system, LNPs have received extensive attention in the treatment of diabetes. In this study, glucose significantly inhibited the *IRS1* expression, and the LNPs at 150 nm significantly improved the expression level of *IRS1* and *INSR*. The expression of *IRS1* mRNA was suppressed by LNPs of other sizes. The high carbohydrate disturbed the insulin signaling pathway, LNPs make it recover to normal levels. The stimulation effect of HG on insulin secretion of largemouth bas*s* was limited, while the 150 nm LNPs could effectively improve the insulin sensitivity of *M. salmoides*.

### 4.4. LNPs Modulate HG‐Induced Gluconeogenesis and Glycolysis

Excess carbohydrates are metabolized by the glycolytic pathway in the liver and inhibit hepatic gluconeogenesis [3]. Activation of gluconeogenesis leads to excessive glycogen accumulation of *M. salmoides*, causing liver damage. The expression of gluconeogenesis‐related genes (*G6Pase* and *FoxO1*) was also increased by HG in *M. salmoides*. Gluconeogenesis in *M. salmoides* was inhibited by the addition of 150 nm of the LNPs, which provided liver protection. Interestingly, 1 h after glucose gavage, similar to the HG group, 175 nm LNPs induced an upregulation of *G6pase* expression, however, at 2 h postgavage, *G6Pase* expression returned to baseline levels. The findings indicate that the velocity of LNCs on bodily processes is influenced by their size. The LNPs at 150 nm restored *GSK* and *GYS1* to normal levels in *M. salmoides* liver. When *GSK* expression is inhibited, the inhibitory effect on glycogen synthase is diminished, thereby facilitating glycogen synthesis [34].

### 4.5. LNPs Modulate PI3K/AKT Signaling Pathway

The PI3K‐AKT signaling pathway is crucial in regulating glucose metabolism [35]. High carbonhydrate feeding resulted in hyperglycemia in *M. salmoides*, subsequently triggering an upregulation in insulin secretion. *PI3K* is activated by elevated insulin levels, leading to upregulated expression. The stimulation of *PI3K* expression may enhance hepatic glucose uptake and glycogen synthesis, while concurrently suppressing fatty acid and protein synthesis [10].

GSK and FoxO1 are pivotal downstream molecules of the PI3K/AKT signaling pathway and are negative regulators of insulin signaling [36]. Stimulation of this signaling pathway can enhance the expression of downstream glucose metabolism genes, such as *GSK3* and *GYS*, facilitating the synthesis and accumulation of substantial hepatic glycogen in *M. salmoides* [37]. In this study, LNPs with sizes of 150 and 175 nm were found to effectively restore the glucose‐induced upregulation of *GCGR*, *PI3K*, and *AKT* mRNA expression levels. This suggests that the LNPs restored gluconeogenesis and reduced hepatic glycogen production in *M. salmoides* hepatocytes. However, it is evident that the recovery rate of LNPs on 150 nm *GCGR*, *PI3K*, and *AKT* mRNA is significantly higher compared to those on 175 nm LNPs. Furthermore, the 200 nm LNPs did not exhibit any restorative effect on *GCGR* mRNA within 4 h, however, they were able to restore the levels of *PI3K* and *AKT* mRNA after the 4 h. This further confirmed that the robust correlation between the size of LNPs and their functionality in terms of velocity.

### 4.6. Limitations

This study has limitations that should be considered. The acute gavage model, while precise, does not fully replicate continuous dietary exposure in aquaculture. Furthermore, the short experimental duration leaves the long‐term metabolic adaptation and efficacy of LNPs unverified. These aspects warrant investigation through future long‐term feeding trials.

## 5. Conclusion

This study provides the first experimental evidence that orally administered LNPs can effectively modulate postprandial glucose metabolism in a carnivorous fish, *M. salmoides*. The regulatory effects were critically dependent on particle size, with 150 nm LNPs demonstrating the most rapid and comprehensive efficacy. Our results indicate that 150 nm LNPs significantly enhanced hepatic insulin sensitivity by upregulating the expression of *irs1* and *insr*. This improvement in insulin signaling was associated with the normalized expression of key genes involved in gluconeogenesis (*foxo1* and *g6pase*) and glycogen synthesis (*gsk3* and *gys1*), effectively counteracting the metabolic disturbances induced by a HG load. Furthermore, the specific upregulation of intestinal *glut5* by 150–200 nm LNPs suggests a potential novel pathway for modulating intestinal glucose sensing. In conclusion, LNPs, particularly of an optimized size, represent a novel and potent nano‐additive strategy with significant potential to improve carbohydrate utilization and metabolic health in carnivorous fish aquaculture.

## Author Contributions

**Kaipeng Zhang**: methodology, validation, writing – original draft. **Tengfei Zhu and Yamin Wang:** validation, formal analysis. **Jing Chen**: conceptualization, methodology. **Shan Xie**: formal analysis. **Zhenye Lin and Xiaotong Chen:** data curation. **Yining Xu**: project administration. **Yingying Yu**: supervision, project administration, funding acquisition.

## Funding

This work was supported by the fund of National Natural Science Foundation of China(Grant 32202911).

## Conflicts of Interest

The authors declare no conflicts of interest.

## Supporting Information

Additional supporting information can be found online in the Supporting Information section.

## Supporting information


**Supporting Information** Figure S1. The LNPs were stored at 4 and 25°C for 30 days, respectively. Lipid nanoparticles (LNPs) with gradient particle sizes of 60 nm, 125, 150, 175 and 200 nm were tested for storage stability in this figure. Freshly prepared LNPs (0 day) were set as the initial control, and the appearance changes of LNPs after 30 days of storage at 4 and 25°C were recorded to visually evaluate the colloidal stability of each formulation. This supplementary data confirmed that the LNPs used in this study maintained good dispersion state and physicochemical stability under conventional storage conditions, which ensured the stability of the test material and the reliability of the in vivo experimental results in the study on largemouth bass (Micropterus salmoides).

## Data Availability

The datasets generated and analyzed during the current study are available from the corresponding author upon reasonable request.
