# Supplementary figures and images for: Size‐Dependent Efficacy of Lipid Nanoparticles in Improving Glucose Utilization of Largemouth Bass (Micropterus salmoides) Under High‐Glucose Conditions
Source: Aquac Nutr. 2026 May 27;2026:7025461. doi: 10.1155/anu/7025461 (PMC13213335; doi:10.1155/anu/7025461)

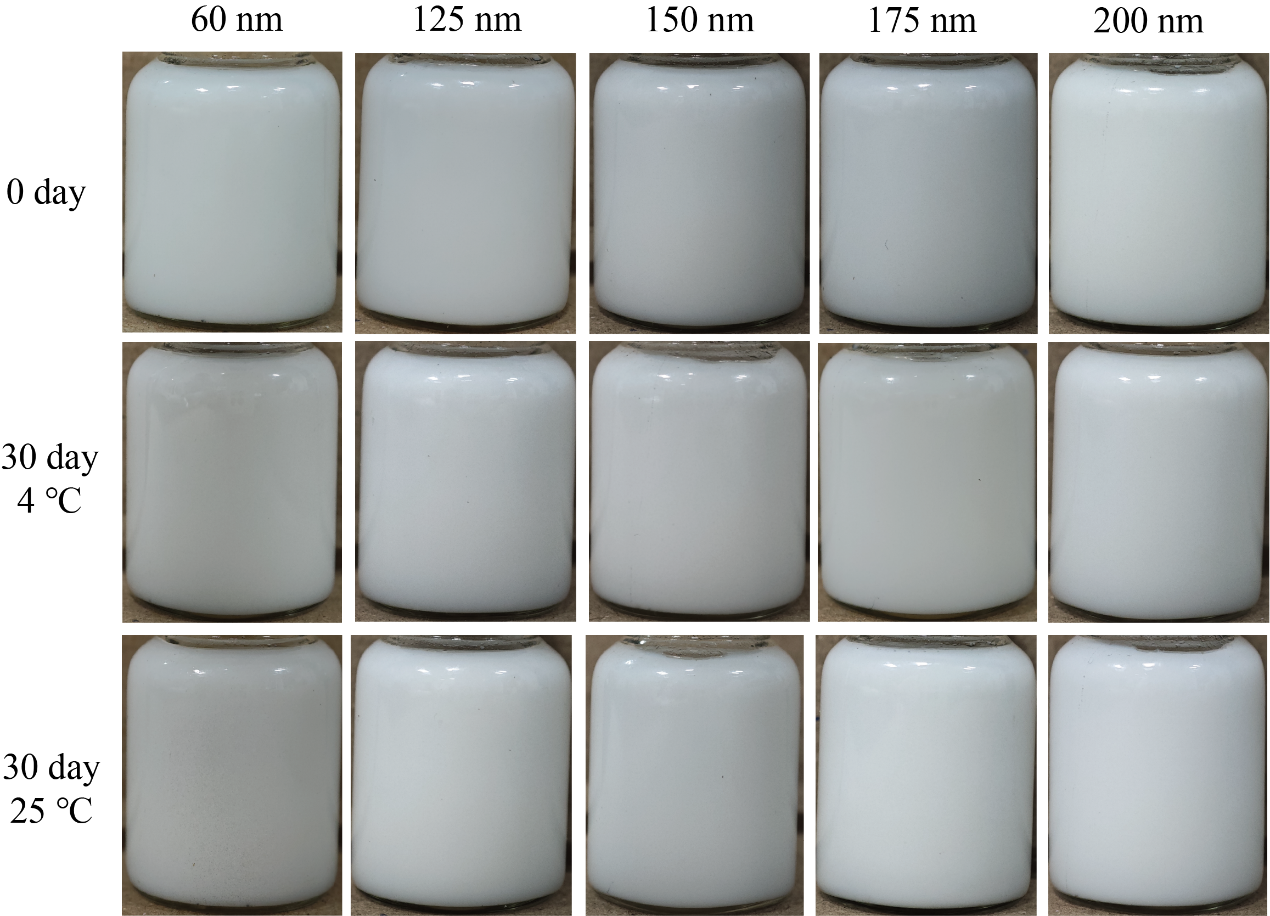


**Figure S1.** The LNPs were stored at 4℃ and 25℃ for 30 days, respectively.

Supplement: Supplementary file 1 — Supporting Information Figure S1. The LNPs were stored at 4 and 25°C for 30 days, respectively. Lipid nanoparticles (LNPs) with gradient particle sizes of 60 nm, 125, 150, 175 and 200 nm were tested for storage stability in this figure. Freshly prepared LNPs (0 day) were set as the initial control, and the appearance changes of LNPs after 30 days of storage at 4 and 25°C were recorded to visually evaluate the colloidal stability of each formulation. This supplementary data confirmed that the LNPs used in this study maintained good dispersion state and physicochemical stability under conventional storage conditions, which ensured the stability of the test material and the reliability of the in vivo experimental results in the study on largemouth bass (Micropterus salmoides). [file ANU-2026-7025461-s001.docx]
